# O041: Overcoming hand hygiene campaign fatigue by an effective innovation involving the infection control link nurses

**DOI:** 10.1186/2047-2994-2-S1-O41

**Published:** 2013-06-20

**Authors:** CWY Cheung, SK Luk, WS Yuen, YY Wong, SS Lau, KH Li, PTY Ching, WH Seto

**Affiliations:** 1 Infection Control Team, Hong Kong Baptist Hospital, Hong Kong, China; 2 Infection Control Team, Hong Kong Baptist Hospital; Pathology Department, Hong Kong Baptist Hospital, Hong Kong, China; 3 Infection Control Team, Hong Kong Baptist Hospital; Pathology Department, Hong Kong Baptist Hospital; WHO Collaborating Centre for Infection Control, Hospital Authority, Hong Kong, China

## Introduction

Hand hygiene was introduced in the 850-bed Hong Kong Baptist Hospital (HKBH), using the promotional techniques recommended by the WHO including a formal kick-off signing ceremony, hospital-wide posters, talks and use of role models. Significant improvement in compliance was observed in 2008 from 41% to 58% (p<0.01). Subsequently from 2009 to 2011, it remained below the 55% level in spite of various promotional activities. This might be related to campaign fatigue.

## Objectives

To improve hand hygiene compliance with the involvement of ICLNs.

## Methods

HKBH has 99 infection control link nurses (ICLNs) in 26 clinical areas and from these focus groups were formed. These identified four key deficiencies and for each a program was implemented to resolve with the help of the ICLNs:

1. **Help your doctors for excellence in Hand Hygiene:** ICLNs reverse the low compliance amongst doctors, the accompanying nurses would squirt alcohol hand rub for them during ward rounds.

2. **Competition for "Speaking Walls" poster:** ICLNs helped their own ward to produce self-made posters, as the present reminders were deemed ineffective. It was believed that self-designed posters would have a better effect.

3. **Identification locations for Point-of-care hand rubs:** ICLNs reported that there were strategic locations without hand rub facilities. 22 such locations were identified.

4. **Hand Hygiene education program and daily checklist for health care assistants (HCAs):** This was implemented by the ICLNs for the reduction in compliance for HCAs is the highest.

## Results

After implementing the four programs, the hand hygiene compliance rate increased to 83% in 2012 (n=1743, CI 81-85%), which is significant (p<0.01) compared to 2009-2011. The alcohol hand rub consumption showed a similar trend in 2012, increasing from 8.1L per 1000 patient-days in 2011 to 9.1L in 2012.

## Conclusion

The strategies described could optimize the end users' participation as not only were ideas extracted from the ICLNs but they also helped in implementing their own ideas. New innovations are vital as Hand Hygiene has been introduced now for a long time and campaign fatigue is likely to occur.

## Disclosure of interest

None declared.

